# Intracellular high cholesterol content disorders the clock genes, apoptosis-related genes and fibrinolytic-related genes rhythmic expressions in human plaque-derived vascular smooth muscle cells

**DOI:** 10.1186/s12944-017-0500-z

**Published:** 2017-07-10

**Authors:** Changpo Lin, Xiao Tang, Lirong Xu, Ruizhe Qian, Zhenyu Shi, Lixin Wang, Tingting Cai, Dong Yan, Weiguo Fu, Daqiao Guo

**Affiliations:** 1 Institute of Vascular Surgery, Department of Vascular Surgery, Zhongshan Hospital, Fudan University, 180 Fenglin Road, Xuhui district, Shanghai, 200032 China; 2 Department of Physiology and Pathophysiology, Fudan University Shanghai Medical College, Shanghai, 200032 China

**Keywords:** Circadian rhythm, Clock-controlled genes, Plaque-derived vascular smooth muscle cells, Cholesterol, Ox-LDL

## Abstract

**Background:**

The clock genes are involved in regulating cardiovascular functions, and their expression disorders would lead to circadian rhythm disruptions of clock-controlled genes (CCGs), resulting in atherosclerotic plaque formation and rupture. Our previous study revealed the rhythmic expression of clock genes were attenuated in human plaque-derived vascular smooth muscle cells (PVSMCs), but failed to detect the downstream CCGs expressions and the underlying molecular mechanism. In this study, we examined the difference of CCGs rhythmic expression between human normal carotid VSMCs (NVSMCs) and PVSMCs. Furthermore, we compared the cholesterol and triglycerides levels between two groups and the link to clock genes and CCGs expressions.

**Methods:**

Seven health donors’ normal carotids and 19 carotid plaques yielded viable cultured NVSMCs and PVSMCs. The expression levels of target genes were measured by quantitative real-time PCR and Western-blot. The intracellular cholesterol and triglycerides levels were measured by kits.

**Result:**

The circadian expressions of apoptosis-related genes and fibrinolytic-related genes were disordered. Besides, the cholesterol levels were significant higher in PVSMCs. After treated with cholesterol or oxidized low density lipoprotein (ox-LDL), the expressions of clock genes were inhibited; and the rhythmic expressions of clock genes, apoptosis-related genes and fibrinolytic-related genes were disturbed in NVSMCs, which were similar to PVSMCs.

**Conclusion:**

The results suggested that intracellular high cholesterol content of PVSMCs would lead to the disorders of clock genes and CCGs rhythmic expressions. And further studies should be conducted to demonstrate the specific molecular mechanisms involved.

## Background

It is well known that the circadian clock genes are involved in regulating physiological and pathological functions of cardiovascular system [1, 2]. Researchers believed the disruption in clock homeostasis would lead to atherosclerotic plaque formation, rupture and subsequent embolism and thrombosis, although the underlying molecular mechanisms were still not clear. The core clock genes include Bmal1, CLOCK, Pers, Crys and Rev-erbα etc., and they affect the diurnal cardiovascular functions by controlling the downstream clock-controlled genes (CCGs). Previous reports illustrated that many apoptosis-related genes, as well as fibrinolytic system factors, which participated in plaque formation and rupture were CCGs, and their circadian rhythms were disordered in apoE knock-out mice [3, 4]. Our previous study found that the rhythmic expressions of clock genes were attenuated, and lipid content was much richer in human plaque-derived vascular smooth muscle cells (PVSMCs) than in normal vascular smooth muscle cells (NVSMCs) [5]. However, we failed to draw the reason for the impairment of clock genes expressions.

In present study, we continued to detect the rhythmic expressions of apoptosis-related genes (Fas, p53 and Bax) and fibrinolytic-related genes (t-PA and PAI-1) in PVSMCs and NVSMCs. Furthermore, as hyperlipidaemia could impair the circadian clock and physiological homeostasis of vascular smooth muscle cells [6], we detected which type of lipid was richer in PVSMCs and whether it affected the clock genes and CCGs circadian rhythms.

## Methods

### Cell culture

NVSMCs and PVSMCs were cultured from normal carotids of healthy donors and carotid plaques by established methods [5]. Seven health donors and 19 patients who underwent carotid endarterectomy between September 2015 and December 2016 in Zhongshan Hospital (Shanghai, China) successfully yielded viable cultured VSMCs. The characteristics of donors and patients were shown in Table 1. The cells were incubated at 37 °C in 5% CO_2_ with the medium changed three times a week. The third to sixth passages of primary cultured cells were used in our study. Two normal carotid samples and four carotid plaques in our study have yielded more smooth muscle cells than others, and their cells were used twice to detect the rhythmic expressions of clock-controlled genes.

**Table 1:** Characteristics of patients succeeded in yielding cultured VSMCs

Type	Number of cases	Gender (M/F)	Age range (mean)	Hypertension	Hyperlipidemia	DM
Human plaque derive VSMCs	19	16/3	48-81(68)	17	3	6
Human normal carotid VSMCs	7	5/2	36-65(47)	2	1	1

*M* male; *F* female; *DM* diabetes mellitus

### Serum shock and cells harvesting

As previously described [5], human VSMCs were seeded in complete medium for 24 h. Then cells were starved for 24 h in serum free medium with or without 50 μg/ml oxidized low density lipoprotein (ox-LDL). Subsequently, the medium was replaced with medium 199 containing 50% horse serum for 2 h. After serum shock, the cells were washed three times with serum free medium and then incubated with starvation medium again until the end of the experiment. The timing of beginning serum shock was defined as Zeitgeber time 0 (ZT0), and cells were harvested every 4 h.

### RNA isolation, complementary DNA preparation and quantitative real-time PCR (qRT-PCR)

Total RNA was extracted from cells using Trizol Reagent (Life Technologies Corporation, USA). Complementary DNA was prepared using the ReverTra Ace qPCR RT Kit (TOYOBO, Japan). RT-PCR was performed on Bio-Rad CFX96™ Real time system using SYBR Green Real-time PCR Master Mix (Bio-Rad) according to the manufacturer’s protocols. The same cycling protocol was used as follows: denaturation at 95.0 °C for 3 min; 40 cycles of 95.0 °C for 15 s, 58.0 °C for 30 s, and 72.0 °C for 15 s + plate read. Glyceraldehyde-3- phosphate dehydrogenase (GAPDH) was used to normalize each mRNA expression level. The mRNA expression levels were presented as relative values in all experiments using the 2^—△△Ct^ formula. The primer sequences of relevant genes were designed by Primer Premier 5 Software and were shown in Table 2.

**Table 2:** The primer Sequences

Gene	GenBank accession	Forward primer (5′–3′)	Reverse primer (5′–3′)
Bmal1	NM_001030272	TGGATGAAGACAACGAACCA	TAGCTGTTGCCCTCTGGTCT
Clock	NM_001267843	CAGAGCACCTTCCCTCAGTC	TTTCCCTCCTTTCCTCAGGT
Per2	NM_022817	CGTGCCAAGCAGTTGACTTA	CAGCAAGGCTCAACAAATCA
Cry1	NM_004075	TAAGAGGCTTCCCTGCAAAA	GCCTCCATTCCCATTAGGAT
Rev-erbα	NM_021724	CTGGGAGGATTTCTCCATGA	TCACTGTCTGGTCCTTCACG
Fas	NM_000043	TATCACCACTATTGCTGGAGTCA	TATCACCACTATTGCTGGAGTCA
p53	NM_000546	GTTCCGAGAGCTGAATGAGG	TCTGAGTCAGGCCCTTCTGT
Bax	NM_004324	CCCGAGAGGTCTTTTTCCGAG	CCAGCCCATGATGGTTCTGAT
PAI-1	NM_000602	CTCTCTCTGCCCTCACCAAC	GTGGAGAGGCTCTTGGTCTG
t-PA	NM_000930	TGGGGAACCACAACTACT	GTGTAAACCTTGCCTATCAG
GAPDH	NM_001256799	GTCAGTGGTGGACCTGACCT	TGCTGTAGCCAAATTCGTTG

### Western-blotting analysis

Cells were lysed with RIPA buffer and then the protein concentration was measured using a BCA protein Assay Kit (Biocolors, CHINA). 50 μg total proteins were separated by 10% SDS-PAGE and transferred onto 0.4 μm PVDF transfer membranes (Millipore, USA). After blocked in 5% non-fat milk for 2 h at room temperature, the membranes were incubation with primary antibodies, including Bmal1 (Cell Signaling Technology, 1:1000),CLOCK (abcam, 1:2000), Rev-erbα (santa-cruz, 1:200), β-actin (VazymeBiotech, 1:10,000) overnight at 4 °C. Then the membranes were washed and incubated with secondary antibody (VazymeBiotech, 1:10,000) for 1 h at room temperature and detected using an enhanced chemiluminescence system (TANON, CHINA). The bands relative intensities were analyzed using Image J software (USA).

### Analysis of cholesterol and triglycerides levels in VSMCs

Cholesterol was extracted from primary VSMCs using the Cholesterol Quantification Kit (Abcam, UK). After extracting using a mixture of chloroform: isopropanol: NP-40 (7:11:0.1), the total cholesterol was measured following the instructions of the kit. Meanwhile, the pellets were lysed with RIPA buffer and the protein concentrations were also measured. The results were presented as μg of cholesterol per mg of cellular protein. The total triglycerides were extracted and measured following the instructions of triglycerides assay kit (Nanjing Jiancheng Bioengineering Institute, CHINA). The results were expressed in mmol of triglycerides per g of cellular protein.

### Statistical analysis

SPSS for Mac, version 21, was used to perform the statistical analysis. Results were demonstrated as mean ± SD. The unpaired student’s t test was used to examine the differences between two groups and two-way analysis of variance (ANOVA) was conducted to evaluate the oscillation of each gene expression. *P* < 0.05 was considered statistically significant.

## Results

### Rhythmic expressions of apoptosis-related genes were irregular in PVSMCs

**Fig. 1:**
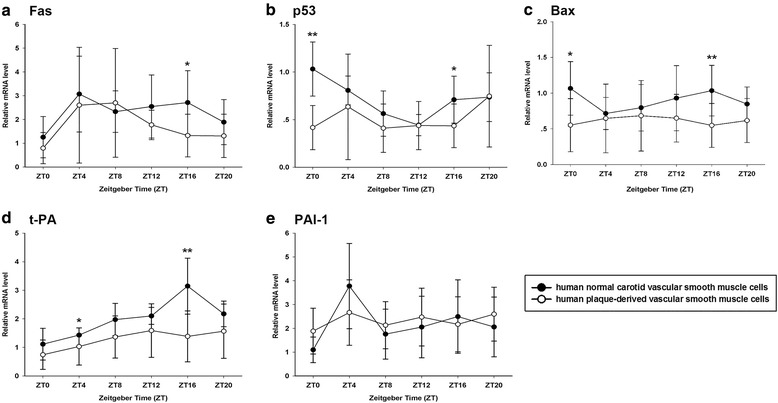
Circadian expressions of Fas, p53, Bax, t-PA and PAI-1 at mRNA levels in NVSMCs and PVSMCs. The mRNA expression levels of Fas (**a**), p53 (**b**), Bax (**c**), t-PA (**d**) and PAI-1 (**e**) were determined by qRT-PCR at indicated time points after serum shock, and were normalized to GAPDH mRNA levels. The signal levels at ZT0 of NVSMCs were defined as 1. Each value was presented as mean ± SD (n_1_ = 9 of NVSMCs samples; n_2_ = 23 of PVSMCs samples). Unpaired student’s t test was used to assess the expression differences between two groups. *p < 0.05 and**p < 0.01 in PVSMCs versus NVSMCs

First, we investigated the mRNA levels of apoptosis-related genes, including Fas, p-53 and Bax (Fig. 1), in order to find out whether their expressions were controlled by clock genes and the potential relationship between them and atherosclerosis. The results revealed that the expressions of Fas and p53 exhibited significant circadian oscillations in NVSMCs (assessed by two-way ANOVA, *p* < 0.05). The expression of Fas peaked at ZT4 and lowest at ZT0.For the mRNA of p53, the peak and trough time was ZT0 and ZT12, respectively. But both of them lost the uniform rhythms in PVSMCs (assessed by two-way ANOVA, *p* > 0.05). Furthermore, the expression level of p53 in PVSMCs was lower than NVSMCs at most of the timing. Bax did not exhibit significant circadian expression in neither groups (assessed by two-way ANOVA, *p* > 0.05).

### The expressions of t-PA and PAI-1 were disordered in PVSMCs

Secondly, we examined the expressions of t-PA and PAI-1 which were associated with thrombosis formation and plaque rupture. It was observed that t-PA and PAI-1 possessed nearly opposite rhythms in NVSMCs (Fig. 1). The expression of t-PA was lowest at ZT0 and gradually increased until reached the peak at ZT16. The expression of PAI-1 was peak at ZT4 then declined sharply and fluctuated with a lower level at other timing. However, their expressions were disordered in PVSMCs. For the mRNA of t-PA, not only the peak time moved to ZT12, but the expression level also reduced in PVSMCs, compared with NVSMCs. The expression of PAI-1 did not illustrate significant combined circadian rhythm in PVSMCs (assessed by two-way ANOVA, *p* > 0.05). In other words, the expression of PAI lost synchronization in PVSMCs. Interestingly, compared with NVSMCs, the level of PAI-1 expression was higher at ZT0, ZT8, ZT12 and ZT20 in PVSMCs.

### The total cholesterol but not triglycerides levels elevated in PVSMCs

**Fig. 2:**
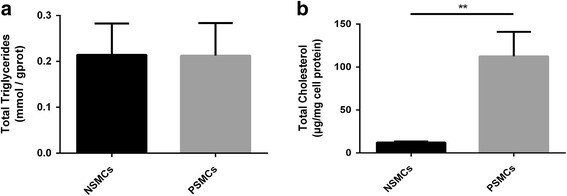
Total triglycerides and cholesterol levels in NVSMCs and PVSMCs. The triglycerides (**a**) and cholesterol (**b**) contents in NVSMCs and PVSMCs were measured by Kits. The data were presented as mean ± SD (*n* = 3). Unpaired student’s t test was used to assess the expression differences between two groups. **p < 0.01 in PVSMCs versus NVSMCs

In our previous study, we found that the lipids contents were much richer in PVSMCs compared with NVSMCs. In order to determine the type of the lipids, we measured the total cholesterol and triglycerides contents in two type of cells. As showed in Fig. 2, the level of total cholesterol was significantly higher in human PVSMCs compared with the normal ones (*p* < 0.01). The total cholesterol contents of human PVSMCs were approximately 112.50 ± 16.45 μg/mg cell protein, while the levels in the controls were 12.18 ± 0.71 μg/mg cell protein, respectively. Meanwhile, the triglycerides contents were almost same between two groups (0.21 ± 0.04 mmol/gprot vs 0.21 ± 0.04 mmol/gprot; *p* = 0.97).

### Cholesterol and ox-LDL inhibited clock genes expressions in the VSMCs

**Fig. 3:**
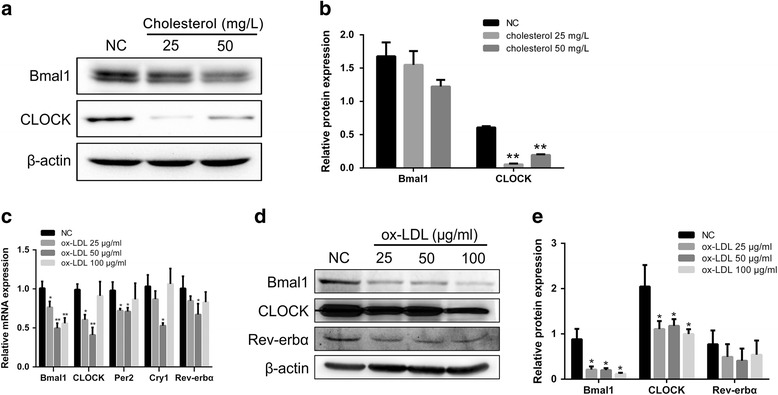
Clock genes expressions in the VSMCs after treated with cholesterol or ox-LDL. **a**. Western-blot analyses of clock genes expressions in VSMCs followed by stimulation with cholesterol for 48 h. **b**. Relative band intensities of Western-Blot, analyzed using Image J software. Each value was normalized to β-actin levels and was presented as mean ± SD (*n* = 3). **c**. Real-time PCR analyses of clock genes expressions in VSMCs followed by stimulation with ox-LDL for 24 h. Each value was normalized to GAPDH mRNA levels and defined the NC levels as 1. Data were presented as mean ± SD (*n* = 3). **d**. Western-blot analyses of clock genes expressions in VSMCs followed by stimulation with ox-LDL for 24 h. **e**. Relative band intensities of Western-Blot, analyzed using Image J software. Each value was normalized to β-actin levels and was presented as mean ± SD (*n* = 3). Unpaired student’s t test was used to assess the expression differences between groups. *p < 0.05 and **p < 0.01 versus NC

As hyperlipidaemia could impair and perturb the periodicity of clock genes in cardiovascular tissues in apoE-deficient mice and VSMCs, we hypothesized the high cholesterol content in PVSMCs might lead to the disturbance of clock gene expression. To test our hypothesis, we treated NASMCs with different concentrations of cholesterol (Sigma) for 48 h. Consistent with our assumption, the expressions of BMAL1 and CLOCK were significantly attenuated after treated with cholesterol (Fig. 3). As LDL is the main cholesterol carrier in the body, we also treated NASMCs with ox-LDL. Both qRT–PCR and Western-blot analysis revealed that the expression levels of main clock genes were significantly decreased upon ox-LDL treatment, too (Fig. 3). The ox-LDL suppressed the clock genes expressions most significant at the dose of 50 μg/ml. Coincidentally, this dose of ox-LDL was often used to mimic in vivo hypercholesterolemia.

### Ox-LDL disturbed the rhythmic expressions of clock genes, apoptosis-related genes and fibrinolytic-related genes in VSMCs

**Fig. 4:**
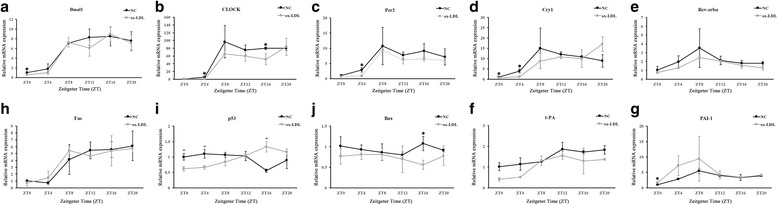
Effects of Ox-LDL on the rhythmic expressions of clock genes and CCGs in the VSMCs. The mRNA levels of Bmal1 (**a**), CLOCK (**b**), Per2 (**c**), Cry1 (**d**), Rev-erbα (**e**), t-PA (**f**), PAI-1 (**g**), Fas (**h**), p53 (**i**) and Bax (**j**) in VSMCs with or without ox-LDL treatment were determined by qRT-PCR at indicated time points after serum shock. Their expression levels were normalized to GAPDH mRNA levels. The signal levels at ZT0 of NC were defined as 1. Each value was presented as mean ± SD (*n* = 6 of each group). Unpaired student’s t test was used to assess the expression differences between two groups. *p < 0.05 versus NC

Furthermore, we examined the rhythmic expressions of clock genes and CCGs mRNA in NVSMCs after stimulated with 50 μg/ml ox-LDL. Similar to PVSMCs, the oscillation amplitudes of clock genes were severely attenuated in ox-LDL treated NVSMCs after serum shock, although they exhibited similar rhythms to normal controls (Fig. 4a-e). Consistently, the circadian expressions of Fas, p53, Bax, t-PA and PAI-1 mRNA were also changed after ox-LDL treatment (Fig. 4f-j). Meanwhile PAI-1 expression was elevated after ox-LDL treatment, too.

## Discussion

In the previous study, we found the expression levels and oscillation amplitude of clock genes were significantly attenuated in PVSMCs compared with NVSMCs [5]. But we failed to figure out the reasons. And we did not detect the expression rhythms of the CCGs through which the core clock genes effort on the diurnal variations of cardiovascular function and the process of atherosclerotic plaque either. In this study, we found that the circadian expressions of apoptosis-related genes and fibrinolytic-related genes were disordered in PVSMCs. Furthermore, we proved the elevated total cholesterol levels in PVSMCs may account at least in part for these changes.

Acute myocardial infarction and stroke are more likely to occur in the early morning. These life-threatening complications of atherosclerosis are mainly due to rupture of plaque, with subsequent embolism, thrombosis and arterial occlusion [7]. Increasing evidence suggests that VSMCs apoptosis could lead to plaque rupture by reducing synthesis collagen isoforms and thinning the fibrous cap [8]. Researchers have identified VSMCs apoptosis in human advanced plaque in vivo [9], with an increasing proportion in unstable lesions [10]. VSMCs isolated from human plaque also exhibit increased apoptosis compared with normal counterparts in vitro. But the factors that impact VSMCs apoptosis in plaques are unclear. It is reported that many apoptosis-related genes are CCGs. In our study, the rhythmic expressions of two apoptosis-related genes, p53 and Fas were changed in human PVSMCs, compared with NVSMCs. Meanwhile, Bax did not exhibit a rhythm neither in NVSMCs nor in PVSMCs. Fas is a member of the TNF receptor superfamily. Previous studies have shown that the disruption of Fas expression would induce the apoptosis of PVSMCs [11, 12]. The role of p53 in atheroma is complex. On one hand, p53 could promote growth arrest, cell senescence and apoptosis within the plaque [13, 14]. On the other hand, some studies revealed that endogenous p53 could also retard trans-differentiation, protect VSMCs against apoptosis and change the mode of cell death in the plaque [15–17]. Consequently, discordant expression rhythms of p53 and Fas in PVSMCs would lead to abnormal cell senescence and apoptosis, which involve in the formation and rupture of carotid plaque.

Human fibrinolytic activity also shows a circadian oscillation, with a trough in the early morning and peak in the afternoon, and that may elicit more frequently onset of cardiovascular events in the morning [18, 19]. PAI-1 is the major inhibitor of fibrinolysis, its expression and activity present a circadian rhythm with a morning peak, which is an antiphase to that of t-PA. We found PAI-1 and t-PA exhibited nearly reverse rhythmic oscillations in NVSMCs in vitro, too. Of note, the level of t-PA expression was reduced, meanwhile the expression level of PAI-1 was elevated in PVSMCs. Previous study also confirmed that the expressions of t-PA and PAI-1 were changed in advanced plaques [20]. It is considered that increased PAI-1 activity is associated with higher risk of cardiovascular events [21, 22]. Moreover, as well as Fas and p53, PAI-1 lost its pooled rhythm while some individuals still possess various rhythmic oscillations in PVSMCs (data not shown). These findings were consistent with previous in vivo studies that their circadian rhythms were disordered in apoE knock-out mice [3, 4], too.

It is well known that the rhythmic expressions of these apoptosis-related genes and fibrinolytic-related genes are controlled by core clock genes like Bmal1, CLOCK, Per2, Cry1 and Rev-erbα [23–25]. So circadian rhythm disorder of these CCGs in PVSMCs should be attributed to the attenuation expression of clock genes we reported previously. Previous studies have confirmed that the hyperlipidaemia could impair the circadian clock in vivo and in vitro [6, 26]. Especially Chen S et al. [6] showed that the free fatty acids (FFAs) could inhibit the clock genes expressions in contractile VSMCs via the suppression of Smarcd1. In this study we revealed that the level of cholesterol, not triglycerides, was significantly elevated in PVSMCs which were most converted to synthetic stages. As we know, the hypercholesterolemia is mainly caused by the increased level of low-density lipoprotein (LDL), as the mutation of LDL receptor or glucokinase gene could lead to dyslipidemia [27, 28]. Then we proved that after treated with ox-LDL, the rhythmic expressions of clock genes were attenuated in VSMCs. Meanwhile, the circadian rhythms of apoptosis-related genes and fibrinolytic-related genes were also disordered, which were similar to PVSMCs. That means, at least in part, the disturbance of clock genes and CCGs rhythmic expressions in PVSMCs were attributed to intracellular high cholesterol content stages. Of course, the atherosclerosis is a complex pathological process. Many other pathophysiological factors may work together to affect the expression levels and rhythms of clock genes and CCGs.

As we know, statins were the most important cholesterol-lowering drugs and their long-term beneficial efforts in reducing cardiovascular morbidity and mortality have been confirmed. But a considerable number of patients throughout the world are intolerant to statins due to the adverse events just like myopathy and hepatotoxicity [29, 30]. Previous study revealed many best-selling and commonly taken drugs (including statins) target genes were rhythmic expressions which controlled by core clock genes [31]. So the circadian clock may affect the statins efficacy and safety pending further study.

Our study had several limitations. Firstly, the number of samples we investigated in current study was quite small. So this were just preliminary results and further we would expand the sample size to confirm the findings. Secondly, the disorders of clock genes and CCGs rhythmic expressions may partly due to potential oxidative stress of ox-LDL. This requires further study to clarify.

## Conclusion

In conclusion, we revealed that the circadian rhythmic expression of the apoptosis-related genes and fibrinolytic-related genes were disordered in PVSMCs. And these changes, together with the decline of clock genes expressions, may partly due to the intracellular high cholesterol content of PVSMCs. Further studies should be conducted to elucidate the specific mechanism underlying molecular links between ox-LDL and clock genes.

## Abbreviations

BAX: BCL2 associated X
Bmal1: Brain and muscle Arnt-like 1
CCGs: Clock-controlled genes
CLOCK: Circadian locomotor output cycles kaput
Cry: Cryptochrome
GAPDH: Glyceraldehyde-3- phosphate dehydrogenase
NVSMCs: Normal vascular smooth muscle cells
ox-LDL: Oxidized low density lipoprotein
PAI-1: Plasminogen activator inhibitor-1
Per: Period
PVSMCs: Plaque-derived vascular smooth muscle cells
qRT-PCR: Quantitative real-time PCR
Rev-erbα: NR1D1 (nuclear receptor subfamily 1, group D, member 1) a member of the nuclear receptor family of intracellular transcription factors
t-PA: Tissue-type plasminogen activator
ZT: Zeitgeber time
